# Continuous Binder-Free Fibers of Pure Imogolite Nanotubes

**DOI:** 10.1021/acsami.1c00971

**Published:** 2021-04-08

**Authors:** Joseph F. Moore, Erwan Paineau, Pascale Launois, Milo S. P. Shaffer

**Affiliations:** † Department of Materials, Imperial College London, Exhibition Road, London SW7 2AZ, U.K.; ‡ Université Paris-Saclay, CNRS, Laboratoire de Physique des Solides, 91405 Orsay, France; § Department of Chemistry, Imperial College London, Exhibition Road, London SW7 2AZ, U.K.

**Keywords:** nanotube, fiber, imogolite, alignment, humidity, jamming, spinning

## Abstract

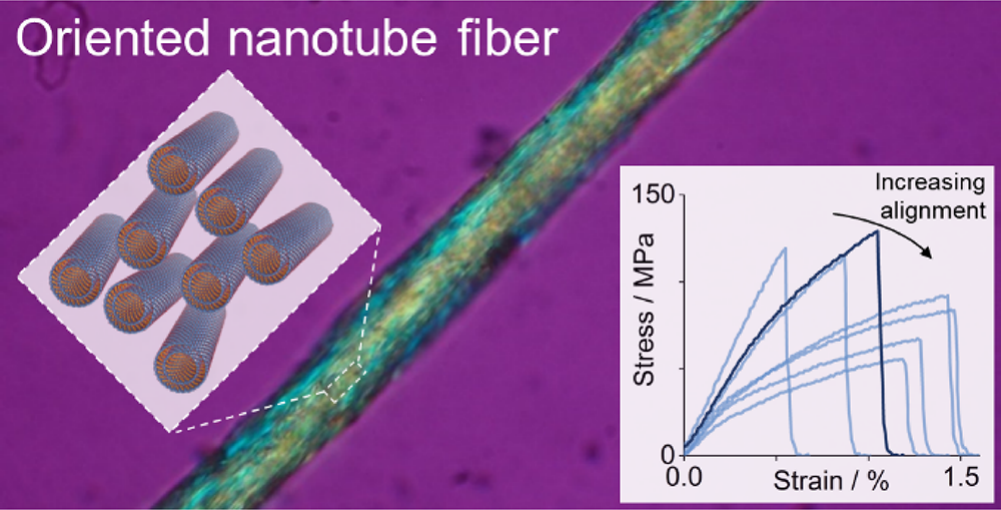

Imogolite nanotubes (INTs) display a range of useful properties and provide an ideal material system to study the assembly of nanomaterials into macroscopic fibers. A method of wet spinning pure, binder-free imogolite fibers has been developed using double-walled germanium imogolite nanotubes. The nanotube aspect ratio can be controlled during the initial synthesis and is critical to the spinning process. Fibers made from short nanotubes (<100 nm) have very low gel strengths, while dopes with longer nanotubes (500–1000 nm) are readily spinnable. The tensile behavior of the resulting imogolite nanotube fibers is strongly influenced by relative humidity (RH), with a modulus of 30 GPa at 10% RH compared to 2.8 GPa at 85% RH, as well as a change in failure mode. This result highlights the importance of inter-nanotube interactions in such assemblies and provides a useful strategy for further exploration. Interestingly, in the absence of a matrix phase, a degree of misorientation appears to improve load transfer between the individual INTs within the porous fiber, likely due to an increase in the number of interparticle contacts. Imogolite nanotubes are an appealing analogue to other nanotube fiber systems, and it is hoped that learnings from this system can also be used to improve carbon nanotube fibers.

## Introduction

Nanotubes and nanorods are promising candidates for producing high-performance fibers due to their excellent intrinsic mechanical properties and compatible one-dimensional morphology. However, the mechanical properties of macroscale fibers are typically significantly lower than those of the constituent nanomaterials due to a combination of processing challenges and weak intermolecular interactions. To date, most studies have focused on carbon nanotube (CNT)-based fibers, using both dry and wet spinning methods;^1−4^ fiber properties have typically been improved by increasing the aspect ratio and alignment of the nanotubes. CNT processing has proven to be particularly challenging given their poor solubility in convenient solvents and tendency to agglomerate. Understanding and optimizing these spinning processes are generally hindered by the difficulty in measuring CNT orientation within the fibers. Strong light absorbance limits the use of optical methods, while polydispersity, irregular packing, and weak scattering make it challenging to study single filament samples with X-rays efficiently.^5^

Imogolite nanotubes (INTs) are an appealing analogue for CNTs due to their monodisperse diameter and spontaneous dispersion in deionized water, which enables simple solution processing.^6^ The archetypal INT is single-walled and comprises a curved outer gibbsite Al(OH)_3_ sheet with isolated O_3_SiOH tetrahedra located inside.^7,8^ However, a variety of INTs can be synthesized via sol–gel methods using different precursors, particularly ones with Ge substituting for Si,^9^ in either a double-walled or single-walled form.^10,11^ Similarly to solutions of CNT polyelectrolytes,^12−14^ INTs form liquid crystalline mesophases, which can be reoriented either by electrical or flow fields,^15,16^ potentially leading to highly aligned fibers. In contrast to CNTs, INT solutions are optically transparent, which greatly aids the use of polarized optical microscopy (POM) in their characterization. Additionally, Al and Ge atoms have much greater X-ray scattering cross sections than carbon atoms, which facilitates the use of lab-source X-rays for studying the structure of both dispersions and macroscale fiber assemblies. The hydrophilicity of INTs also enables the tuning of nanotube interactions by varying humidity. INTs have been shown to adsorb up to 80% of their weight in water at 95% relative humidity (RH)^17^ with the water adsorbed in the internal cavity, interstitially between the walls of double-walled nanotubes and crucially on the outer surface.^18^ In principle, by varying the humidity, the strength of inter-nanotube hydrogen bonding should be modified, enabling the influence of shear interactions on the mechanical properties of nanotube-based fibers to be studied.^19,20^

In addition to their appeal as a model system, INTs are increasingly of interest in their own right, with potential applications in molecular filtration, catalysis, and molecular transport and release.^6^ While INTs have lower absolute mechanical properties than CNTs, the strength and stiffness are still significant, with a predicted elastic modulus between 100 and 400 GPa.^21−24^ More generally, they have complementary properties to CNTs in electrical conductivity, color, and hydrophilicity. Nanocomposites and hybrid films of INTs with polymers and biomaterials have previously been produced,^25−27^ and recently, continuous INT-polyvinyl alcohol composite fibers have been prepared that show a degree of self-healing at remarkably high absolute strengths.^28^ However, as yet, no macroscale fibers have been reported from pure INTs. This study, therefore, investigates the possibility of using wet spinning to create pure, binder-free imogolite nanotube fibers and explores the effects of nanotube length, alignment, and hydration on fiber processability and mechanical properties.

## Synthesis of DW Ge-INTs

**Figure 1:**
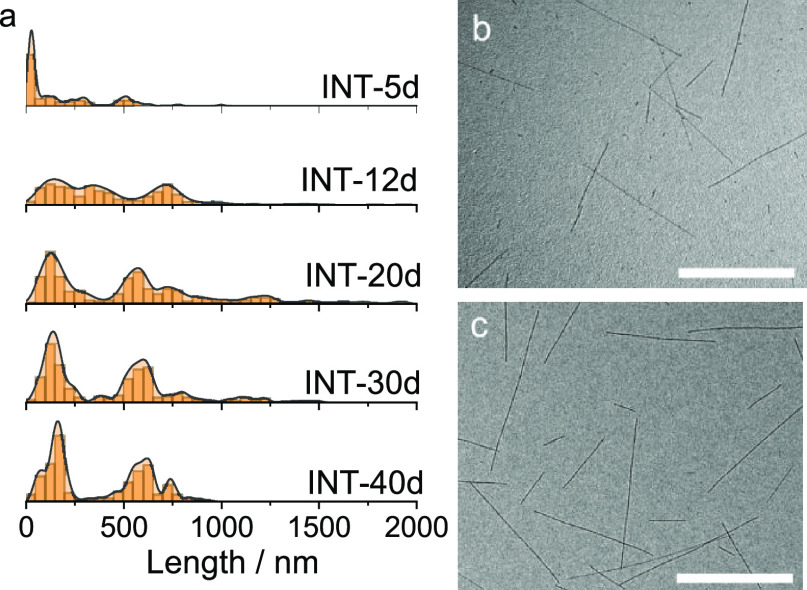
(a) Length number distributions of INT feedstocks determined by measurements in TEM (illustrative distribution curves have been generated with a Gaussian kernel density estimator). (b,c) Representative TEM images of (b) INT-5d and (c) INT-40d. Scale bars are 500 nm.

It is challenging to spin fibers from nanomaterials such as CNTs and nanocellulose if the aspect ratio is too low.^29,30^ Therefore, in order to enhance the spinnability in this work, the INT synthesis was developed to provide high-aspect-ratio feedstocks. Compared to the conventional synthesis route using sodium hydroxide,^31^ the in situ production of hydroxyl ions by thermal decomposition of urea produces significantly longer double-walled germanium imogolite nanotubes (DW Ge-INTs) after 5 days of reaction; the nanotube inner and outer diameters remain consistently 1.6 and 4.3 nm, respectively.^32^ Although several studies have been devoted to the mechanisms of nanotube growth during synthesis,^33,34^ the effect of long reaction times (>5 days) remains largely unexplored. In this work, the aging time of the nanotube synthesis was varied from 5 to 40 days (feedstocks INT-5d, INT-12d, INT-20d, etc.) to create spinning dopes with an expected further increase in length. The length distribution of each INT feedstock was determined by measuring the length of at least 200 nanotubes over several TEM images (Figure 1).

INT-5d is dominated by short nanotubes (<100 nm). With further incubation, these nanotubes were consumed to produce a bimodal distribution with average lengths centered at 150 and 600 nm. Beyond 20 days of incubation, little further change to the distribution was identified. Oriented attachment (OA) of formed nanotubes has previously been proposed as a key mechanism of INT growth after consumption of the initial precursors and topological rearrangment of proto-imogolite nanostructures.^33,35^ However, this growth mechanism is expected to occur extremely slowly at larger nanotube lengths due to the *L*^–4^ dependency of tip–tip collision frequency, explaining the lack of meaningful further growth after 20 days.^36^ IR spectroscopy indicates no significant difference in the chemical bonding within the INTs for the different reaction periods (Figure S1).

## Spinning DW Ge-INT Fibers

Binder-free imogolite fibers were spun by injecting an aqueous spinning dope solution of DW Ge-INTs (9 g/L) through a needle into a coagulation bath of calcium chloride in water (300 g/L). The spinning dopes exhibited strong birefringence (Figure S2), indicative of an ordered mesophase observed previously in both carbon and imogolite nanotube dispersions,^16,37,38^ which should be ideal for liquid crystalline wet spinning. The high ionic strength of the coagulant (*I* = 8.1 M) enabled the quick gelation of the spinning dope. At slow injection rates, a transparent gel monolith formed at the tip of the needle. Increasing the injection velocity led to the formation of a gel-like protofiber that could be manipulated and then collected on a winding wheel to provide drawing during the spinning process (Supplementary Movie 1). The fracture strength of the protofibers, and thus their ease of manipulation, depended strongly upon the length distribution of the nanotube feedstock. The longer nanotube feedstocks (12d–40d) could be easily spun, whereas frequent filament breakage occurred with the INT-5d feedstock. It was observed that the filament breakage consistently occurred at the coagulant/air interface, indicating that the surface tension of the coagulant liquid imparts sufficient force to overcome the gel strength of the fiber. The stress in a cylindrical fiber being withdrawn from a liquid is inversely proportional to its radius (Figure S3). Thus, increasing the radius of the gel fiber should reduce the probability of filament breakage. In accordance with this theory, the INT-5d feedstock was found to be unspinnable using a 24 gauge needle (internal diameter, ID, of 311 μm) but spinnable using a 21 gauge needle (ID of 514 μm). The gel strength of the INT-5d protofiber was therefore estimated to be approximately 500 Pa. The other feedstocks, from INT-12d to INT-40d, were successfully drawn from the coagulant without breakage using a 27 gauge needle (ID of 210 μm), implying a gel strength of at least 1.5 kPa. These gel strengths are relatively low, requiring careful handling of the protofibers before collection and drying. Polymer/INT hybrid hydrogels typically have significantly higher fracture strengths; for comparison, polyacrylamide hydrogels with a 5% INT loading have been produced with a strength of 220 kPa,^39^ and hyaluronic acid/INT hydrogels with 1 and 10% INT loadings have shown strengths of 20 and 100 kPa, respectively.^40^ Unlike polymers, the rigid rod INT system is held together only by friction and jamming rather than molecular entanglement.

**Figure 2:**
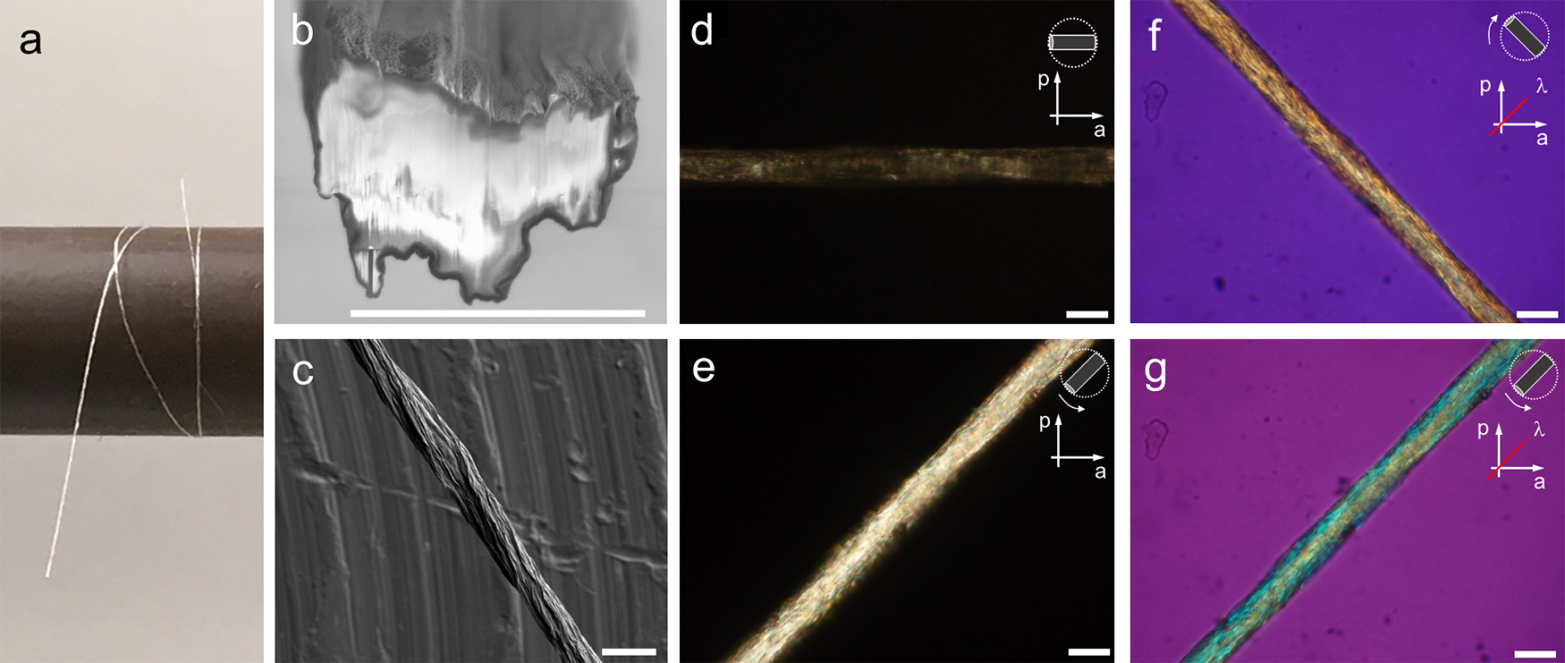
(a) Photograph of a fiber section on a spool, (b) SEM image of a focused ion beam-milled cross section of a Au-coated dry fiber, (c) SEM image of a Au-coated dry fiber on an Al stub, (d–g) polarized optical microscopy images for fibers from INT-20d without (d,e) and with (f,g) a retardation plate (λ plate, 530 nm). The orientations of the polarizer and analyzer are indicated by labels p and a, respectively, while the red line represents the slow axis of the retardation plate λ. The fiber was tilted by +/–45° with respect to the horizontal direction as indicated in the upper right corner. Scale bars are 20 μm.

After washing in deionized water and drying in ambient conditions for 48 h, the resulting INT fibers were robust and readily handled with an average diameter of ∼20 μm, textured surfaces, and irregular cross sections typical of nanomaterial fibers^41^ (Figure 2a–c and Figure S4). Energy-dispersive X-ray spectroscopy indicates that the dry fibers are mostly composed of aluminum, germanium, and oxygen with small residues of calcium (3 at. %) and chlorine (6 at. %) remaining after the washing procedure (Figure S4). As expected from the Ge-INT structural formula (GeAl_2_O_7_H_4_), the measured atomic ratio of aluminum to germanium was 2:1. Although it is challenging to image the internal structure by SEM due to the low electrical conductivity and small INT diameter, the fiber microstructure appears uniform with no evidence of a skin-core texture. The packing density of the fibers can be estimated from the dope concentration, needle area, draw ratio, fiber cross-sectional area, and density of a DW Ge-INT (Supplementary Note 1). This estimate yields packing densities of around 45–55%, similar to that of floating catalyst chemical vapor deposition CNT fibers (∼35 to 60%)^42^ but lower than highly optimized wet spun CNT fibers (∼75%).^29^

Polarized optical microscopy (POM) observations indicate a strong birefringence in all fibers, with a maximum of transmission observed with the fiber axis at 45° to the crossed polarizers (Figure 2d,e and Figure S5). The use of a retardation plate of 530 nm gives rise to different interference colors depending on the orientation of the fiber relative to the slow axis of the λ plate and shows that the nanotube axes are aligned with the fiber axis (Figure 2f,g).

**Figure 3:**
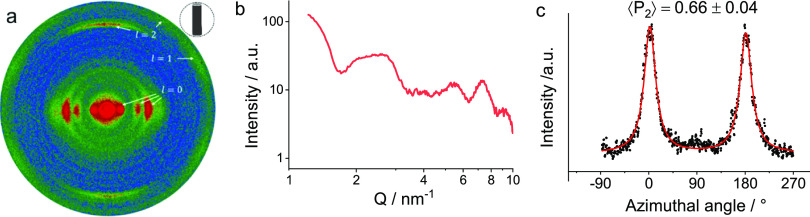
(a) False color X-ray scattering pattern of a dry fiber from INT-20d, (b) radial profile of scattered intensity in a sector ±30° from the equatorial line after geometric and polarization corrections, and (c) azimuthal profile of scattered intensity at 2.6 nm^–1^ with a Lorentzian fit and the value of ⟨*P*_2_⟩ calculated from the direct space ODF.

X-ray scattering (XRS) patterns were used to quantify the orientation within the fibers (a typical example is shown in Figure 3a); in these pure fibers, all the observed scattering features can be attributed to the INTs. The intensity scattered by a single nanotube is located in reciprocal planes perpendicular to its long axis, at , where *T* is the period of the nanotube atomic structure along its long axis (*T* ≈ 8.5 Å) and *l* is an integer. In these patterns, the scattering features from the fibers are modulated angularly with the *l* = 0 scattering signal located on the equator of the fiber scattering pattern and the (002) feature centered in the fiber direction, indicating the preferred orientation of the nanotubes along the fiber axis.^28^ The intensity on the *l* = 0 line (Figure 3b and Figure S7) exhibits oscillations characteristic of the squared form factor of DW Ge-INTs.^32^ Although the broad modulation around 2.6 nm^–1^ is flattened, the lack of sharp Bragg peaks indicates that any local structural organization is limited to bundles of a small size.^43,44^ The formation of larger, more ordered bundles is presumably hindered during the fast coagulation process, given the relatively large size and mass of the INTs (∼2–17 MDa), compared to typical polymer molecules that may crystallize. The angular distribution of the *l* = 0 signals is used to quantitatively determine the orientation of nanotubes within the fibers.^28,45,46^ The angular distribution of scattering intensity in reciprocal space fits well to a Lorentzian distribution (Figure 3c and Figure S8), which, when transformed to an orientation distribution function (ODF) in direct space, results in a Lorentzian function to the power 1.5.^28^ The Hermans order parameter, or ⟨*P*_2_⟩, is used to characterize the orientation between perfectly uniaxial (⟨*P*_2_⟩ = 1) or randomly oriented (⟨*P*_2_⟩ = 0); it was calculated from the direct space ODF as , where φ is the angle between the long axis of the nanotube and the fiber. The calculated values range from 0.49 to 0.74 for the eight fiber samples studied by XRS (Table S1). Measurements performed at varying positions along the length of a single fiber section indicate that the degree of alignment is constant within each section; the differences in the orientation between samples are likely to arise from the manual handling of each fiber section.

## Mechanical Properties of DW Ge-INT Fibers

**Figure 4:**
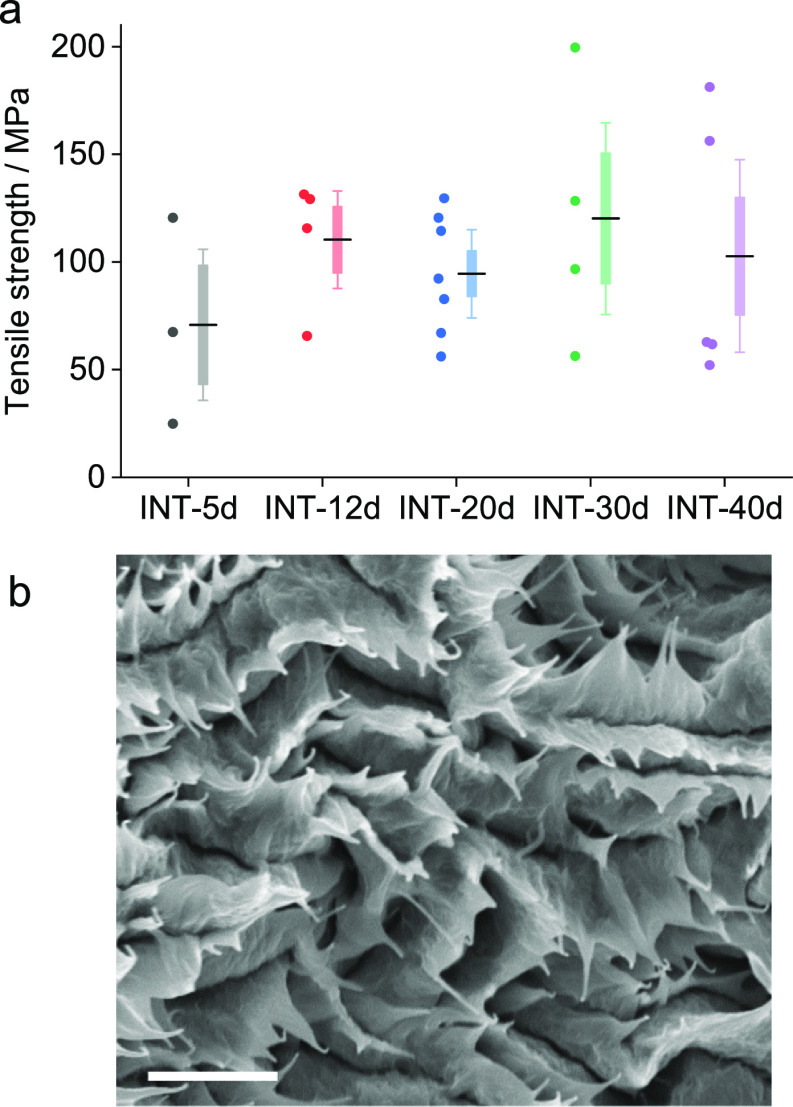
(a) Tensile strength of fibers for each feedstock (dots show individual test results, dashes show the mean value, and boxes and whiskers show the standard error and 90% confidence interval for the mean) and (b) SEM image of the fracture surface of INT fibers showing a pull-out failure. The scale bar is 1 μm.

The mechanical properties of the pure INT fibers were measured using a conventional single filament tensile test. The tensile strength of dry fibers across the INT feedstocks follows the same trend observed in their gel strengths, with a higher strength for feedstocks INT-12d to INT-40d and a lower strength for INT-5d. The data is quite scattered for these small batch fibers. However, within error, the strengths and stiffnesses of fibers produced from feedstocks INT-12d to INT-40d were similar with an ultimate tensile strength and elastic modulus in ambient conditions (40% RH) of approximately 100 MPa and 10 GPa, respectively (Figure 4a). The specific properties of the fibers can be determined using the breaking force (10–50 mN) and estimated linear density (∼0.4 tex) with a corresponding tenacity of 0.02–0.12 N tex^–1^. A lower failure stress was measured for the shorter INT-5d sample (∼60 MPa). These values are similar to other networks of hydrophilic nanoscale elements such as cellulose nanofibers (tensile strength of ∼10–500 MPa and elastic modulus of ∼2–30 GPa^30,47^) and to carbon nanotube fibers assembled from surfactant dispersions^48^ but lower than highly optimized, denser carbon nanotube fibers spun from liquid crystalline mesophases, which can have tensile strengths exceeding 4 GPa. In addition to the increased intrinsic strength and modulus, the significantly higher strengths of the optimized CNT fibers may be due to the much larger aspect ratio of the nanotubes. The best performing CNTs^49^ had an aspect ratio of around 6700, while the INTs in this work had an aspect ratio of ∼200. An approximately linear relationship between the fiber tensile strength and CNT aspect ratio has been reported previously,^29^ as might be expected below the critical stress transfer length in a discontinuous nanotube assembly.^50^ In this regime, the fibers fail by shear slip of nanotubes, as illustrated by electron micrographs of fracture surfaces showing a “finger-like” pull-out structure (Figure 4b). This fibrillar failure, due to nanotube slip, has previously been noticed in carbon nanotube fibers with a similar packing density.^1,51^

**Figure 5:**
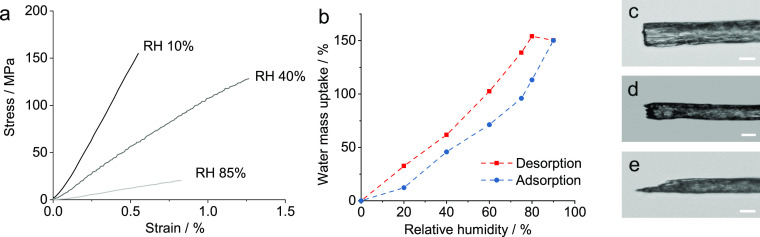
(a) Stress–strain curves for fiber samples of INT-30d at controlled humidity, (b) dynamic vapor sorption isotherms at 25 °C showing water uptake with relative humidity for adsorption and desorption cycles, and (c–e) optical micrographs of INT-30d fibers fractured at (c) 10, (d) 40, and (e) 85% relative humidity. Scale bars are 20 μm.

The importance of shear interactions in determining the fiber mechanical properties is illustrated by the effect of humidity (Figure 5a). The modulus, in particular, is strongly dependent upon the humidity during the test, with a modulus of 30 GPa at 10% RH compared to 2.8 GPa at 85% RH. Due to the strongly hydrophilic nature of both the INTs and remnant CaCl_2_, increasing humidity leads to significant adsorption of water, with the uptake reaching 150% by mass at 90% RH (Figure 5b and Figure S6). The presence of water is expected to modify the interactions between the surface hydroxyl groups, leading to a reduction in both the shear strength and shear modulus of inter-nanotube interactions, without affecting the intrinsic INT axial properties. The 10-fold decrease in the tensile strength and modulus with increasing humidity therefore indicates that the fiber properties are driven by inter-nanotube interactions, which are weakened by incorporation of water into the fiber structure. The change in failure mode of these INT fibers with relative humidity is clearly shown by the fracture surfaces (Figure 5c–e). Fibers tested at low relative humidity show a flat brittle fracture surface. As humidity is increased, more pullout is evident, and at high relative humidity, local necking and shear deformation are visible. The humidity dependence is broadly reversible, with fibers conditioned at 85% RH and then tested at 40% RH showing similar tensile behavior to those simply conditioned at 40% (Figure S9).

**Figure 6:**
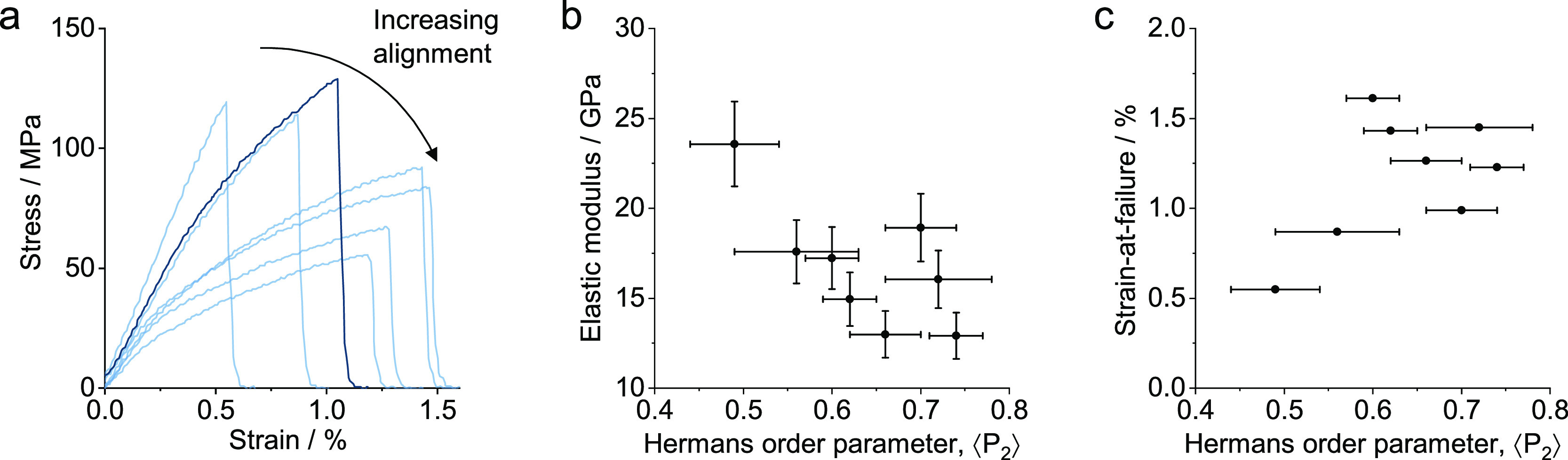
(a) Stress–strain curves of fiber samples from INT-20d, (b) elastic modulus, and (c) strain-at-failure as a function of the Hermans order parameter.

In order to explore the scatter in fiber properties in more detail, a larger number of tensile samples were tested for INT-20d. The stress–strain curves exhibit the typical shape seen in nanomaterial fibers. There is a short initial take-up related to the straightening of fibers followed by an elastic region and then plastic deformation characteristic of internanotube slip,^52^ indicated by a prominent “elbow” at around 25 MPa in most of the cases (Figure 6a). Perhaps surprisingly, higher degrees of alignment correlate strongly with a lower elastic modulus and a higher strain-to-failure (Figure 6b,c). This result initially seems to be counterintuitive, particularly when considering typical short-fiber composite models, which predict increased stiffness and strength with increased alignment.^53,54^

However, this trend may be explained by considering the microstructure and load transfer within these relatively porous fibers in the absence of any matrix. The fibers are comprised of stiff, straight rods, which have a low interfacial shear strength. In highly aligned fibers, the load is transferred between the nanotubes by relatively mobile point contacts, which can slide without arrest, leading to ductile behavior and a higher strain-to-failure. However, in less aligned packings of high-aspect-ratio rods, the load can also be supported by mechanical interlocking, which occurs through a jamming transition when the number of independent contacts per rod exceeds a critical value of around 10.^55,56^ This mechanism of load transfer has been observed in systems across a variety of length scales including bird nests, bamboo skewers, and colloidal gels.^57−59^ As the number of contacts between rigid rods increases with increased misorientation,^60^ it is expected that jamming occurs more frequently in the less aligned INT fibers and leads to more efficient stress transfer within the fiber and hence a higher modulus. The deformation of these fibers may be considered as analogous to the shear of granular assemblies of frictional rigid rods. Such assemblies are modeled to undergo extensive shear alignment in order to reduce interparticle contact and reduce the stress required for deformation.^61^ As the solid volume fraction of these models increases above 50%, a rapid increase in the shear stress occurs due to mechanical percolation through the formation of rigid clusters. We propose that the INT fibers are in this mechanical percolation regime, with a percolation threshold that increases with alignment.^62^ At a constant volume fraction, within this regime, less oriented systems are more networked. The granular modeling also highlights the importance of friction in determining the extent of network formation, which here correlates with the effects of humidity on mechanical response.

The orientational dependence is more manifest in these INT fibers than in analogous CNTs or nanocellulose fibers due to the comparably short aspect ratio and high rigidity of the INTs. The individual INTs are less able to bend and form the necessary contacts for frictional load transfer and do not kink or fibrillate in the manner of CNT and nanocellulose bundles. The coagulation/gelation process delivers porous fibers near the percolation threshold, and as a result, the jamming behavior is the most important to the fiber mechanical properties. At higher packing fractions, or with a composite matrix,^28^ a more conventional increase in mechanical properties with alignment can be anticipated.

## Conclusions

Continuous macroscopic fibers of pure imogolite nanotubes can be prepared by lab-scale wet spinning, which, as a form of conventional coagulation spinning, can be readily optimized and scaled up. In the future, these binder-free fibers can be converted into more complex macroscale constructs by weaving, braiding, or other textile processes. They may find applications in catalysis or molecular filtration by providing a robust structure that enables reagent access to monodisperse nanotubes and their nanoscale pores. In addition, once optimized, INT fibers may be used as reinforcements in structural composites. While the absolute performance will be lower than that of carbon nanotube-based fibers, the improved surface interaction with the matrix, oxidation stability, and optical transparency may offer advantages, just as glass fibers are often used in preference to carbon fibers in many composite applications. The ease of fiber spinning and the resulting fiber properties are both strongly linked to the length distribution of the INT feedstocks. Short INTs, less than 100 nm, were challenging to spin due to the low gel strength of the protofibers. However, longer INTs formed more robust gels, as well as stronger and stiffer dried fibers. The formation of a liquid crystal mesophase in the spinning dope likely contributes to the alignment of the INTs due to shear during spinning. However, in contrast to typical models of nanotube yarns, fiber strengths and stiffnesses were lower for more aligned fibers. This unusual finding can be attributed to the relatively low aspect ratio of the INTs and their high rigidity, which makes them much straighter than equivalent CNTs or biological nanofibrils and less able to bend to form contacts. They are, therefore, much less likely to entangle or interlock in aligned fibers. Combined with relatively low-strength intertube interactions, which are easily disrupted by ambient water, misorientation is needed to transfer the load across the individual INTs within the porous fiber. Ductile failure occurs first due to frictional sliding of the INTs followed by pullout. The most significant factor currently affecting the mechanical properties of these INTs fibers is the relative humidity during tensile testing. Reducing the humidity removes the water from the intertube space, suppressing the plasticity associated with INT sliding. As a result, the tensile strength and modulus of the fibers increase dramatically from 20 to 155 MPa and 2.7 to 30 GPa, respectively, when reducing humidity from 85 to 10% RH. This result highlights the importance of controlling shear interactions in nanomaterial fibers in general. The INT fibers provide a useful model system in which the shear interactions can be systematically explored through humidity. Through this system, an optimized relationship between the aspect ratio, alignment, and interfacial shear properties may be identified, which can then be implemented in other, more demanding, material systems. With further optimization of the feedstock and spinning process, denser, stronger INT fibers may usefully complement CNT fibers in structural and multifunctional composites.

## Methods

### Synthesis of DW Ge-INTs

Double-walled Ge-INTs were synthesized using aluminum perchlorate nonahydrate (reagent grade, Alfa Aesar), tetraethoxygermane (TEOG, ≥99.95%, Sigma-Aldrich), and urea (>99%, Sigma-Aldrich) following the procedure described elsewhere.^32^ TEOG was mixed in a PTFE beaker with an aqueous solution of aluminum perchlorate (*C* = 0.2 mol L^–1^) and a urea solution with a molar ratio of [Ge]:[Al]:[urea] = 1:2:2. After mixing, the PTFE beaker was placed in an acid digestion bomb (Zeoclave, Maximator, France) for hydrothermal treatment at 140 °C. The solution was recovered after 5 days and then dialyzed against ultrapure water using semipermeable membranes (Spectra/Por, cutoff = 10 kDa) until the conductivity of the bath drops below 0.5 mS m^–1^.^37^

### Preparation of INT Fibers

INT fibers were prepared by injecting each INT feedstock through a 21 gauge needle at 5 mL/h (linear velocity of 6.7 mm s^–1^) into a coagulation bath of aqueous calcium chloride (300 g L^–1^). The fibers were collected on a rotating PTFE wheel (diameter of 100 mm and surface linear velocity of 33.5 mm s^–1^) with a spin draw ratio of 5. After collection on the wheel, the fibers were cut into 15 cm lengths and washed by dipping in deionized water (3 × 2 s). A small tag of aluminum foil (∼20 mg) was attached to one end of each fiber, and they were hung to dry in ambient conditions.

### Tensile Testing of INT Fibers

INT fiber samples were tested following the standard BS ISO 11566:1996. Fiber samples were mounted onto card frames with a gauge length of 15 mm with the ends fixed with an epoxy adhesive (Araldite Rapid, Huntsman Advanced Materials, Ltd., GB). The tensile tests were conducted on a TST350 tensile stress tester (Linkam Scientific Instruments, Ltd., GB) with a 2 N load cell and a crosshead speed of 1 mm min^–1^. The cross-sectional area for each sample was determined using the observed diameter in transmission optical microscopy.

### Characterization of INT Fibers

A small portion of INT feedstock was sampled and dried for further infrared (IR) characterization between KBr pressed pellets (∼1 wt % dry DW Ge-INT powder). IR spectra were acquired in transmission mode (Nicolet iS50) by averaging 256 scans at a resolution of 4 cm^–1^. Transmission electron microscopy (TEM) was performed on highly dilute dispersions of a feedstock (1 mg L^–1^) prepared in ethanol and deposited in a carbon-coated copper grid. TEM micrographs were recorded with a JEOL 1400 operating at 80 kV. X-ray scattering (XRS) experiments were carried out on a rotating anode (model RU H3R, Rigaku Corporation, JP) using Cu Kα radiation (λ = 0.154 nm) delivered by a multilayer W/Si optics. Pieces of single fibers were mounted on cardboard struts and placed in a beam path. Some samples were imaged in a lab atmosphere (for which a background due to scattering from air was subtracted), and others were imaged using a vacuum chamber equipped with a collimator, with entrance and exit windows made of 500 mm-thick Mylar films. The fiber was kept perpendicular to the incident X-ray beam. Two-dimensional patterns were recorded on a MAR345 detector (marXperts GmbH, DE) with 150 μm pixel size. Extraction of the scattered intensity *I* as a function of the scattering modulus *Q* (*Q* = 4π sin θ = λ, where 2θ is the scattering angle) or azimuthal angle τ was performed with home-developed software. Scanning electron microscopy (SEM) and energy-dispersive X-ray spectroscopy (EDX) were carried out using a JEOL 6010LA microscope on Au sputter-coated samples at an accelerating voltage of 20 kV. Cross sections were imaged using a Zeiss Auriga CrossBeam focused ion beam scanning electron microscope after milling with a 30 kV Ga^+^ ion beam at a beam current of 30 nA. The water sorption behavior of INT fibers was investigated through the dynamic vapor sorption (DVS) technique using a DVS Advantage apparatus (Surface Measurement Systems, UK). Several strands of INT fibers (∼13 mg) were manually shaped into a ball of sample and loaded in an aluminum pan. The moisture sorption was analyzed over a range of preset RH conditions starting from 0% RH and increasing to 90% RH in different RH steps at 25 °C before decreasing to 0% RH to obtain the desorption data. The moisture sorption measurements were recorded using DVS Analysis Suite software.

## Supporting Information Available

Infrared spectra of DW Ge-INTs, crossed polarizer optical observations of aqueous DW Ge-INT suspensions, schematic of the modified Wilhelmy method, SEM images of focused ion beam milled cross sections and energy-dispersive spectroscopy of INT fibers, crossed polarizer optical images of INT fibers, dynamic vapor sorption isotherm of DW Ge-INTs, radial profiles of scattered X-ray intensity, azimuthal profiles of scattered X-ray intensity, stress–strain curves of fibers tested at 40% RH with and without initial conditioning at 85% RH, table of structural parameters and mechanical properties, and note on the calculation of packing density (PDF)
Video of the spinning process using a take-up wheel (MOV)

## Supplementary Material

am1c00971_si_001.pdf

am1c00971_si_002.mov
