# Supplementary material for: Continuous Binder-Free Fibers of Pure Imogolite Nanotubes
Source: ACS Appl Mater Interfaces. 2021 Apr 8;13(15):17940–7. doi: 10.1021/acsami.1c00971 (PMC8153543; doi:10.1021/acsami.1c00971)
Supplement: Supplementary file 1 — am1c00971_si_001.pdf [file am1c00971_si_001.pdf]

Supporting Information

Continuous Binder-free Fibers of Pure Imogolite Nanotubes

Joseph F. Moore,[1] Erwan Paineau,[2] Pascale Launois,[2,] Milo S. P. Shaffer[1,3,*]*

[1] Department of Materials, Imperial College London, Exhibition Road, London, SW7 2AZ, UK

[2] Université Paris-Saclay, CNRS, Laboratoire de Physique des Solides, 91405 Orsay, France

[3] Department of Chemistry, Imperial College London, Exhibition Road, London, SW7 2AZ, UK

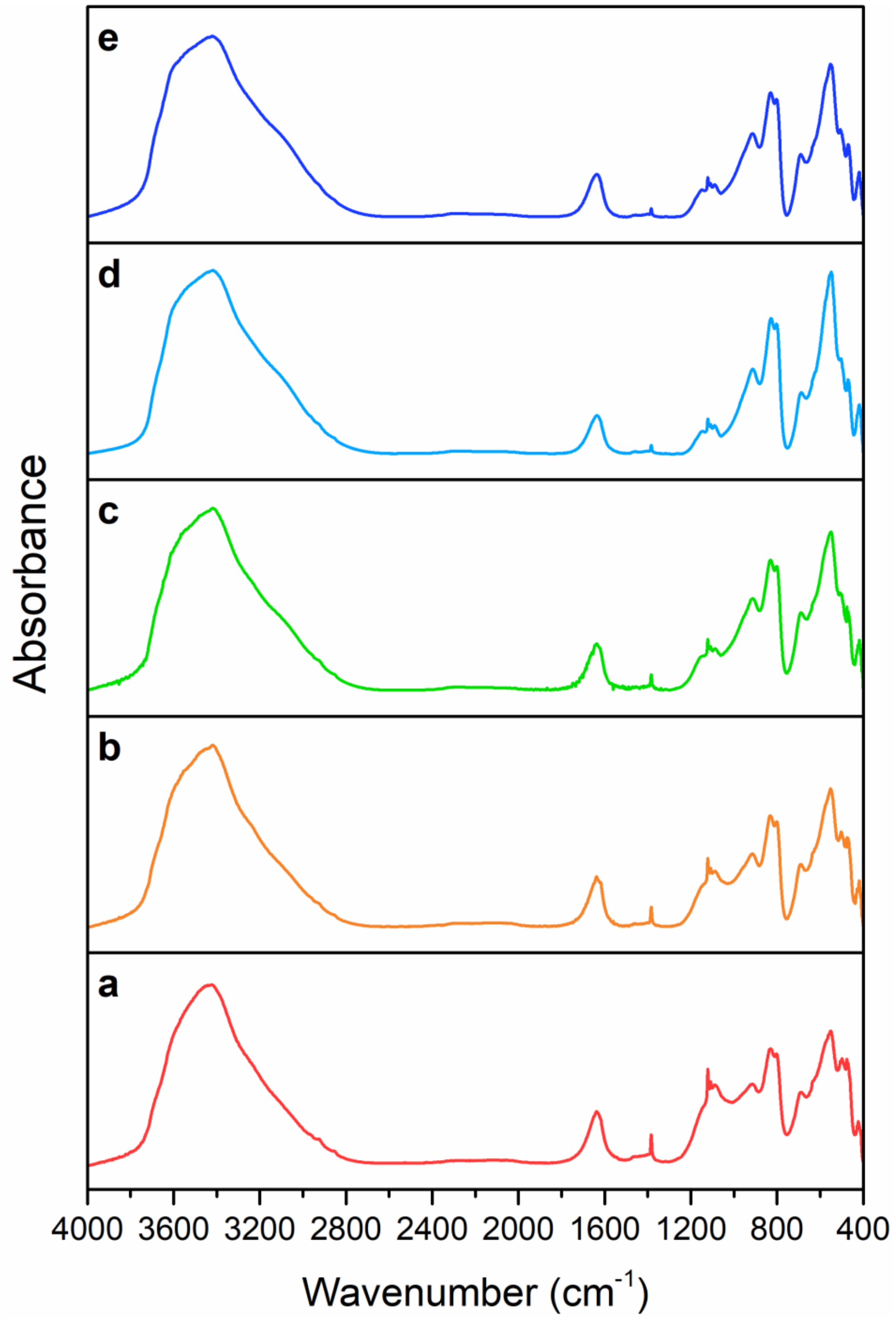

Figure S1. Infrared spectra of DW Ge-INTs as a function of the growth time. (a) 5 days, (b) 12 days, (c) 20 days, (d) 30 days, (e) 40 days. Peaks at 1635 cm^{-1} correspond to H_2O bending mode. Vibrational bands at 915, 835 and 805 cm^{-1} are related to Ge-O stretching and the broad peak around 555 cm^{-1} corresponds to Al-O stretching.

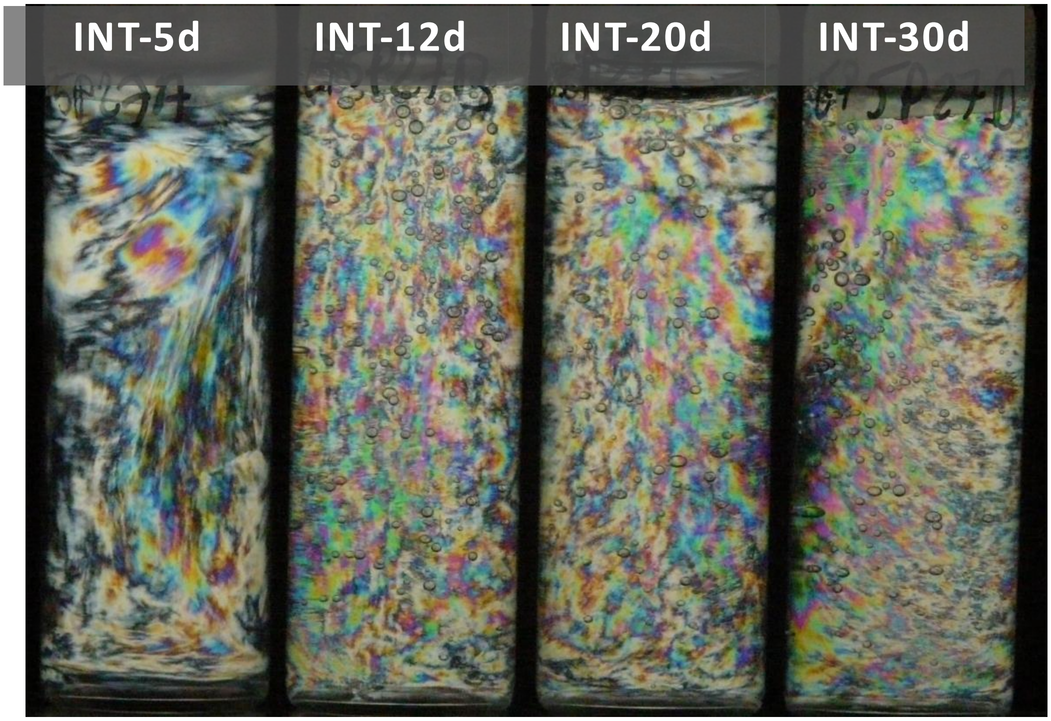

Figure S2. Optical observations between crossed polarizers of aqueous suspensions of DW Ge-INTs produced with different synthesis times

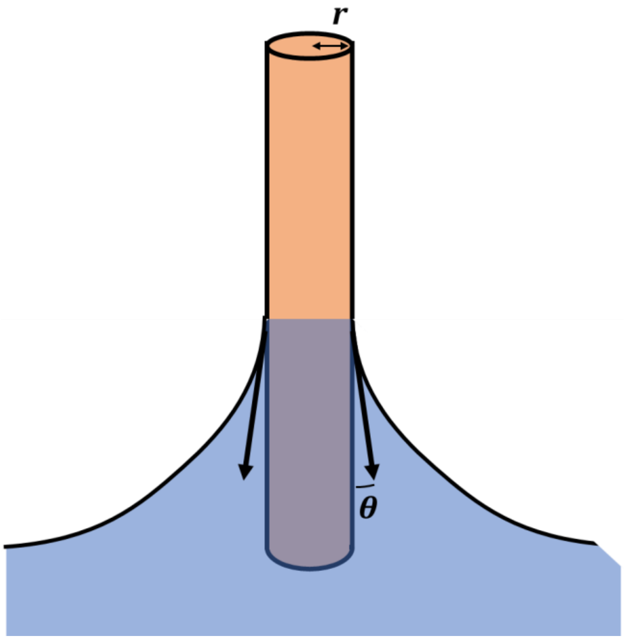

Figure S3. Illustration of modified Wilhelmy method to estimate fiber gel strengths

The fiber gel strengths were estimated by considering the force acting on the fiber due to the surface tension in a modified Wilhelmy arrangement. The stress within the fiber is given by:

$$\sigma = \frac{F}{A} = \frac{2\pi r\, \gamma\, cos\theta}{\pi r^2} = \frac{2\, \gamma\, cos\theta}{r} \qquad \text{(S1)}$$

where γ is the coagulant surface tension, r is the diameter of the gel fiber and θ is the angle between the fiber axis and the coagulant surface at the point of contact.

Taking the approximation that $cos\theta \sim 1$, the stress within the fiber during extraction from the coagulant is:

$$\sigma \sim \frac{2\gamma}{r} \qquad (S2)$$

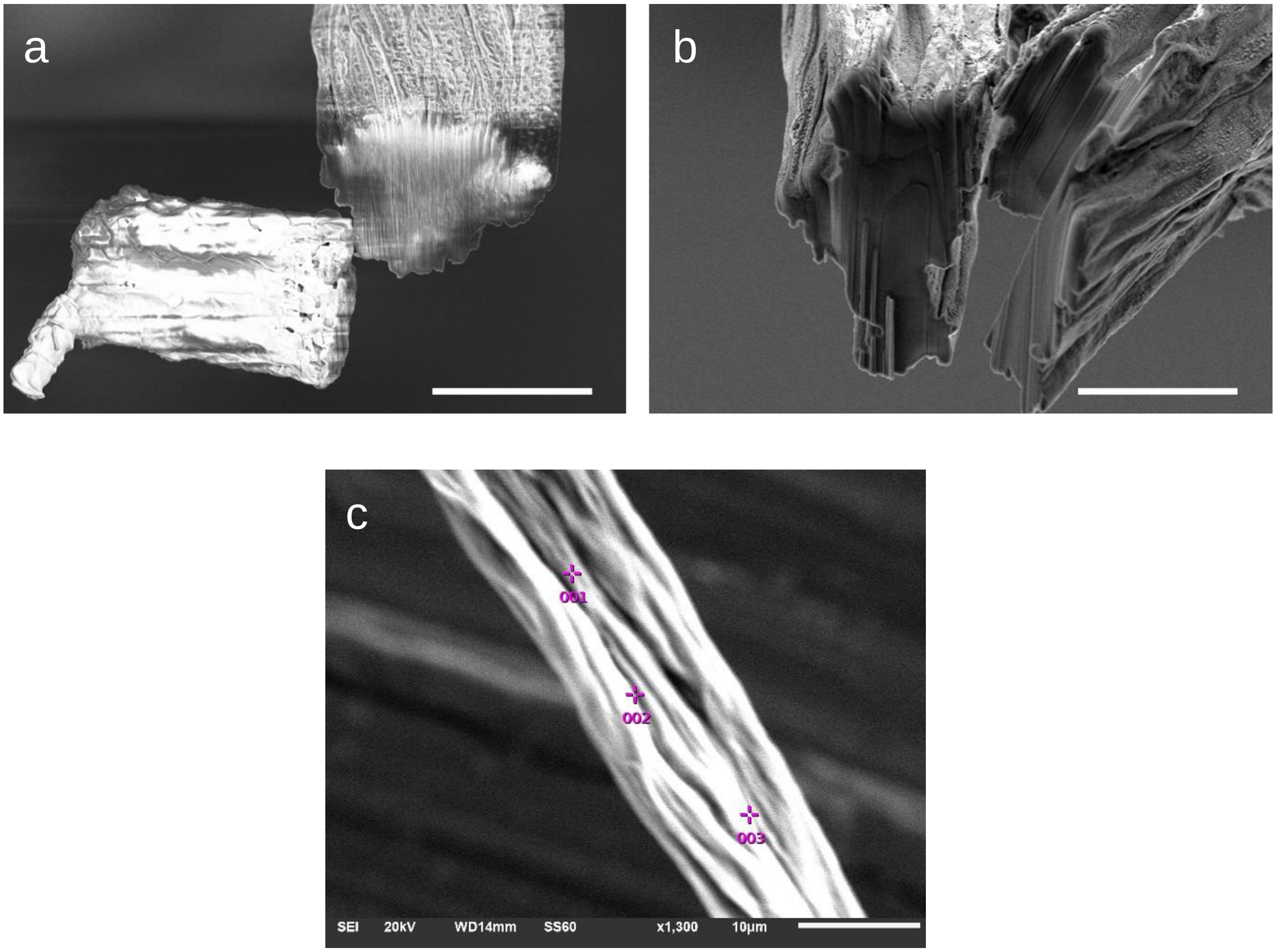

Spot	O / at %	Al / at %	Ge / at %	Ca / at %	Cl / at %
001	62.1	20.8	8.7	2.9	5.4
002	60.3	21.1	9.8	3.3	5.6
003	59.5	21.4	9.8	3.4	5.9
Average	60.6	21.1	9.4	3.2	5.6

Figure S4. (a,b) SEM images of focused ion beam milled cross-sections of Au-coated dry fibers, (c) SEM image of fiber with corresponding elemental composition as calculated by energy dispersive spectroscopy. Scale bars 10 µm.

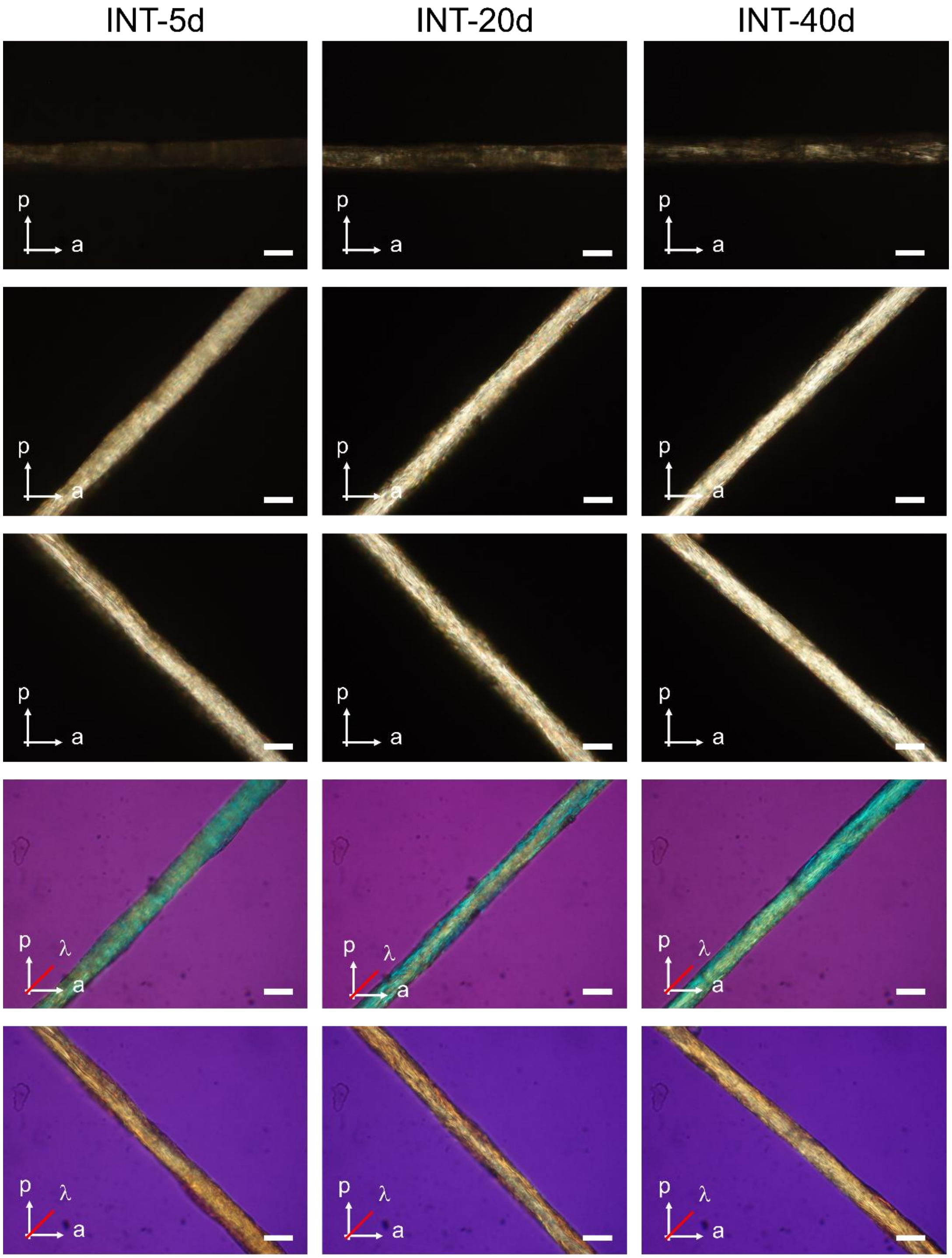

Figure S5. Polarized optical microscopy images of fibers from INT-5d, INT-20d and INT-40d without and with retardation plate (λ-plate, 530 nm). The orientation of the polarizer and analyzer are indicated by labels p and a, respectively, while the red line represents the slow axis of the retardation plate λ. Scale bars 20 μm.

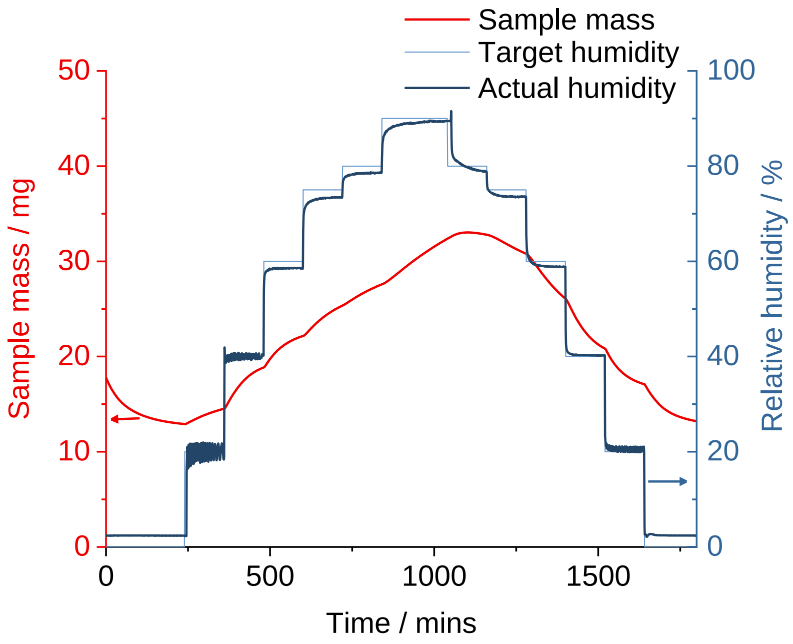

Figure S6. Dynamic vapor sorption isotherm at 25°C showing sample mass as a function of time with varying relative humidity.

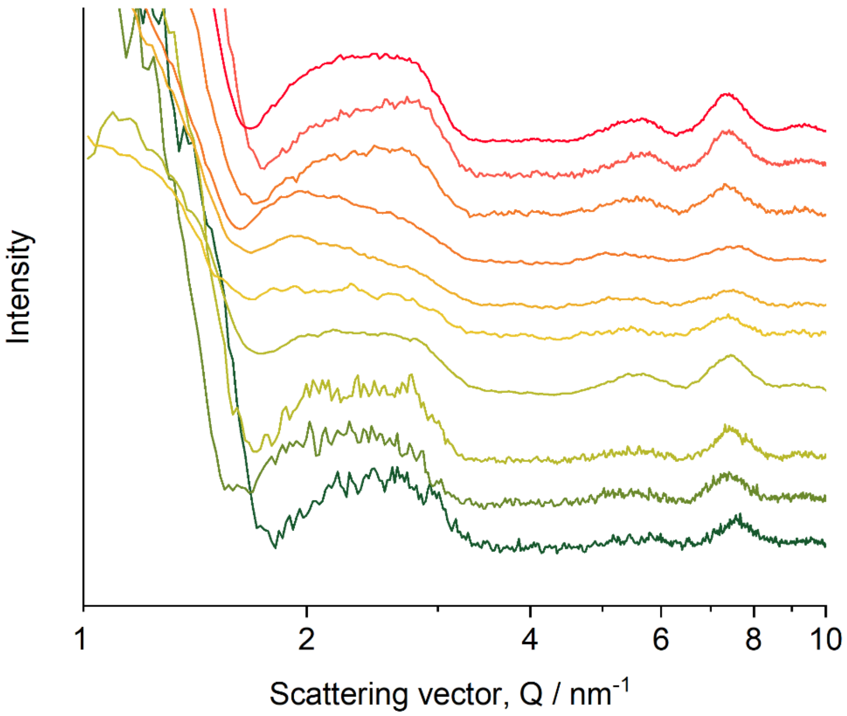

Figure S7. Radial profiles of scattered X-ray intensity (after geometric and polarization corrections) in a sector ± 30° from the equatorial plane showing the characteristic oscillations of DW Ge-INTs. Lines have been translated vertically for clarity and intensities are on a logarithmic scale.

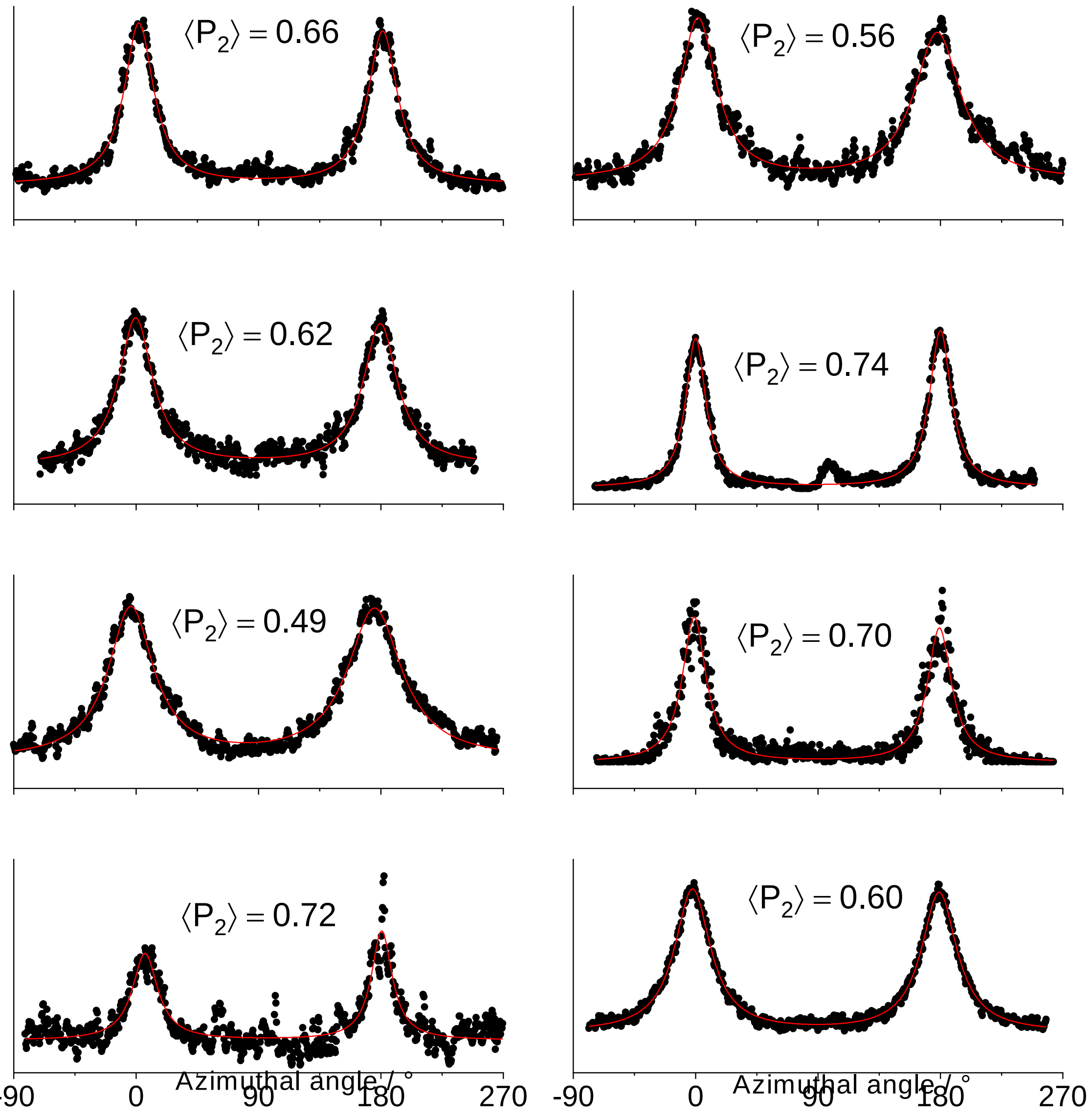

Figure S8. Azimuthal profiles of scattered X-ray intensity at 2.6 nm^{-1} with Lorentzian fits and values of $\langle P_2 \rangle$ calculated from direct space ODF.

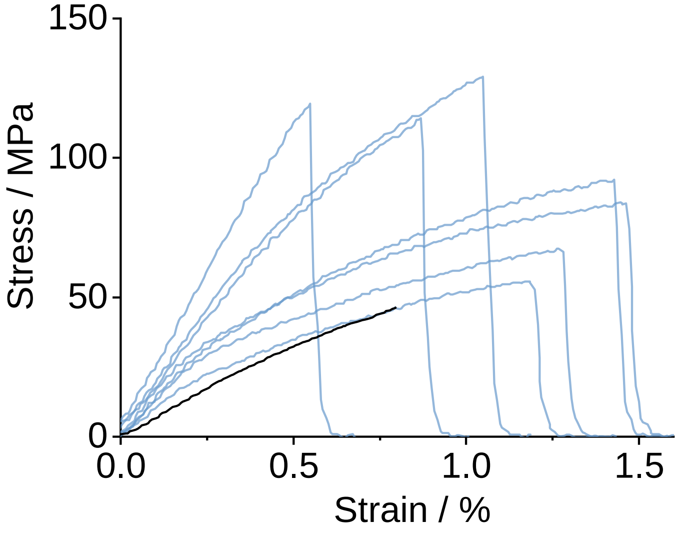

Figure S9. Stress strain curves of fibers from INT-20d tested at 40% RH with no prior conditioning (light blue). Similar results were obtained for samples initially conditioned at 85% RH for two hours (black), before re-equilibrating and testing at 40% RH.

Table S1. Structural parameters and mechanical properties of individual fiber samples used for mechanical testing with an index to identify each sample.

Sample ID	$\langle P_2 \rangle$	Tensile strength / MPa	Strain-at-failure / %	Elastic modulus / Gpa
INT-20d,2	0.66 ± 0.04	67	1.26	13.0
INT-20d,3	0.72 ± 0.06	84	1.45	16.0
INT-20d,4	0.56 ± 0.07	114	0.87	17.6
INT-20d,5	0.49 ± 0.05	119	0.55	23.7
INT-20d,7	0.62 ± 0.03	92	1.43	14.9
INT-12d,2	0.70 ± 0.04	129	0.99	18.9
INT-30d,3	0.74 ± 0.03	128	1.23	12.9
INT-40d,4	0.60 ± 0.03	153	1.61	17.2

Supplementary Note 1

The packing density of the INT fibers has been estimated using the volumetric dope injection rate Q, the dope mass concentration c (mass of dry INTs per unit volume of the aqueous dope), the needle area A, the spin-draw ratio DR, the dry fiber diameter d, and the density of a DW Ge-INT ρ_{INT}. The density of a DW Ge-INT was calculated using the density of the nanotube walls (3.6 g cm^{-3})[1] and the outer diameter (4.34 nm), internal diameter (1.54 nm) and nanotube wall

thickness (0.6 nm)[2] to give ρ_{INT}=2.7 g cm^{-3} as the mass of a nanotube divided by the volume of the cylinder given by its outer diameter.

The volume of INTs injected in time τ, V_{INTs} is given by:

$$V_{INTs} = \frac{Q\ c\ \tau}{\rho_{INT}}$$

The volume of fiber produced in time τ, V_{fibre} is given by:

$$V_{fibre} = \frac{\pi\ d^2}{4} \times DR\ \left(\frac{Q\ \tau}{A}\right)$$

The resulting packing density is given by:

$$Packing\ density = \frac{V_{INTs}}{V_{fibre}} = \frac{4\ c\ A}{\pi d^2\ \times \rho_{INT} \times DR}$$

1. Lee, W. J.; Paineau, E.; Anthony, D. B.; Gao, Y.; Leese, H. S.; Rouziere, S.; Launois, P.; Shaffer, M. S. P., Inorganic Nanotube Mesophases Enable Strong Self-Healing Fibers. *ACS Nano* **2020,** *14,* 5570-5580.

2. Amara, M. S.; Paineau, E.; Bacia-Verloop, M.; Krapf, M. E.; Davidson, P.; Belloni, L.; Levard, C.; Rose, J.; Launois, P.; Thill, A., Single-Step Formation of Micron Long $(OH)_3Al_2O_3Ge(OH)$ Imogolite-Like Nanotubes. *Chem Commun (Camb)* **2013,** *49*, 11284-6.
